# Investigating the Interplay between Myeloma Cells and Bone Marrow Stromal Cells in the Development of Drug Resistance: Dissecting the Role of Epigenetic Modifications

**DOI:** 10.3390/cancers13164069

**Published:** 2021-08-13

**Authors:** Jacqueline Schütt, Theresa Nägler, Tino Schenk, Annamaria Brioli

**Affiliations:** 1 Clinic of Internal Medicine 2, Hematology and Oncology, Jena University Hospital, 07747 Jena, Germany; Jacqueline.schuett@med.uni-greifswald.de (J.S.); Theresa.Naegler@med.uni-jena.de (T.N.); tino.schenk@med.uni-jena.de (T.S.); 2 Institute of Molecular Cell Biology, Center for Molecular Biomedicine Jena (CMB), Jena University Hospital, 07747 Jena, Germany; 3 Clinic of Internal Medicine C, Hematology and Oncology, Stem Cell Transplantation and Palliative Care, Greifswald University Medicine, 17475 Greifswald, Germany

**Keywords:** multiple myeloma, epigenetics, bone marrow microenvironment

## Abstract

**Simple Summary:**

Despite advances made in the last two decades, multiple myeloma (MM) is still an incurable disease. The genetic complexity of MM and the presence of intra-clonal heterogeneity are major contributors to disease relapse and the development of treatment resistance. Additionally, the bone marrow microenvironment is known to play a pivotal role in MM disease progression. Together with genetic modifications, epigenetic changes have been shown to influence MM development and progression. However, epigenetic treatments for MM are still lacking. This is mainly due to the high rate of adverse events of epigenetic drugs in clinical practice. In this review, we will focus on the role of epigenetic modifications in MM disease progression and the development of drug resistance, as well as their role in shaping the interplay between bone marrow stromal cells and MM cells. The current and future treatment strategies involving epigenetic drugs will also be addressed.

**Abstract:**

Multiple Myeloma (MM) is a malignancy of plasma cells infiltrating the bone marrow (BM). Many studies have demonstrated the crucial involvement of bone marrow stromal cells in MM progression and drug resistance. Together with the BM microenvironment (BMME), epigenetics also plays a crucial role in MM development. A variety of epigenetic regulators, including histone acetyltransferases (HATs), histone methyltransferases (HMTs) and lysine demethylases (KDMs), are altered in MM, contributing to the disease progression and prognosis. In addition to histone modifications, DNA methylation also plays a crucial role. Among others, aberrant epigenetics involves processes associated with the BMME, like bone homeostasis, ECM remodeling or the development of treatment resistance. In this review, we will highlight the importance of the interplay of MM cells with the BMME in the development of treatment resistance. Additionally, we will focus on the epigenetic aberrations in MM and their role in disease evolution, interaction with the BMME, disease progression and development of drug resistance. We will also briefly touch on the epigenetic treatments currently available or currently under investigation to overcome BMME-driven treatment resistance.

## 1. Multiple Myeloma and Its Microenvironment

Multiple myeloma (MM) is the second-most frequent hematologic malignancy. It is characterized by the clonal expansion of monoclonal plasma cells [1]. In the last years, many novel targets for specific MM treatment have been identified. A better understanding of MM biology translated in the widespread use of drugs with novel mechanisms of action, such as immunomodulatory drugs (IMiDs, namely thalidomide, lenalidomide and pomalidomide) and proteasome inhibitors (PIs, namely bortezomib, carfilzomib and ixazomib). These compounds, together with the most recent implementation in the clinical praxis of the anti-CD38 monoclonal antibodies (MoAbs) daratumumab and isatuximab, and the anti-SLAMF7 MoAb elotuzumab, have significantly improved both the progression-free survival (PFS) and overall survival (OS), with a median OS that, in some studies, is now approaching 10 years [2,3,4,5,6,7,8,9,10]. Nevertheless, despite the progresses made, MM is still an incurable malignancy, and patients will eventually develop treatment resistance and succumb to their disease [11]. The bone marrow microenvironment (BMME) plays a pivotal role in myeloma progression and in the development of disease resistance. In MM, the bone marrow is posing as a niche consisting of microenvironmental cellular components (stromal cells, osteoblasts, osteoclasts, adipocytes, endothelial cells, T cells and natural killer cells), the extracellular matrix (ECM, laminins and fibronectin) and adhesion molecules (e.g., syndecan-1, VCAM1 and VLA4). Cytokines and growth factors (e.g., IL-6, TNF-α, HGF and IGF) are released within the bone marrow niche by both stromal and plasma cells, providing ideal conditions for MM cell survival, growth and the development of drug resistance [12,13].

MM development is a multistep process. During this process, not only the genetic complexity of the disease increases [14] but, also, the complexity of its microenvironment. An increased activation of osteoclasts; an increase in angiogenesis and an altered expression of growth factors, cytokines and adhesion molecules are all hallmarks of MM disease progression [15]. Drug resistance is also widely attributed to the interplay between MM cells and the BMME [16].

There exist three essential mechanisms of how the BMME promotes the growth, expansion and survival of MM cells: (1) the release of soluble factors, (2) direct cell-cell contacts (3) and the production of extracellular vesicles/exosomes [16,17]. 

The release of exosomes by both MM bone marrow mesenchymal stromal cells (BM-MSC) and MM cells has been shown to induce MM cell growth. Exosomes derived from the BM-MSC of MM patients contain oncogenic proteins, adhesion molecules and cytokines (such as IL-6 and CCL2) important for the expansion of MM cells. These exosomes also lack the tumor-suppressive microRNA 15a (miR-15a), further promoting MM cell growth. Interestingly, exosomes derived from normal BM-MSC have an inhibitory effect on MM cells, supporting the notion that, in MM, the BMME is also, in some form, affected by the hematologic disease [18].

A major soluble player contributing to the protective effect of the BMME on MM cells is IL-6. IL-6 can mediate the upregulation of telomerase activity via PI3K/Akt/NF-κB [19]. Additionally, IL-6 stimulates osteoclastogenesis and inhibits osteoblastogenesis [20], thus interfering with bone homeostasis and fostering the development of MM bone disease. The interplay of MM cells with bone homeostasis is regulated via many factors, including osteoclast stimulating factors (MIP-1α, RANKL, VEGF, TNF-α, IL-1β, HGF and IL-6) and osteoblast inhibitory factors (IL-3, IL-7 and the Wnt pathway inhibitor DKK1) [13,21]. The adhesion of MM cells to the stroma promotes the secretion of the above-mentioned factors, thus increasing MM cell survival and fostering the development of MM bone disease [12]. 

Drug resistance can be caused by the adhesion of MM cells to stroma cells and to the extracellular matrix, the so-called cell adhesion-mediated drug resistance (CAM-DR), or can be mediated via the release of cytokines and chemokines, the so-called soluble factor-mediated drug resistance (SFM-DR) [22]. Both mechanisms have been extensively studied. In CAM-DR, the adhesion molecule VLA4 plays a major role in the homing to the BMME, as well as in the development of chemoresistance [12]. In addition, PSGL-1 has been shown to be crucial for the adhesion and homing of MM cells to the bone marrow microenvironment [23]. The adhesion of MM cells to the cells of the bone marrow downregulates Fas and upregulates 3-survivin (a caspase-3 inhibitor), leading to the induction of a cell adhesion-mediated immune resistance against T-cell cytotoxicity [24]. Another important player in CAM-DR are macrophages that, by inhibiting BCL-xL-dependent caspase activation, prevent drug-induced apoptosis [25].

SFM-DR can be mediated by the AP-1 family member JunB. The interaction of MM with the BMME upregulates JunB through the release of IL-6, even in the absence of direct cell–cell contact. JunB regulates the genes involved in apoptosis, DNA replication and metabolism, thus inducing drug resistance [26]. Additionally, the paracrine release of sonic hedgehog by MM cells induces an upregulation of CYP26 and reduces retinoid signaling within the BMME, subsequently inducing a protective effect by establishing a B-cell-like, bortezomib-resistant phenotype [27].

## 2. Multiple Myeloma Epigenetics

### 2.1. DNA Methylation

As stated, MM is defined by its clonal diversity, which complicates the application of genetically targeted therapy [28]. What is more, not only genetic modifications but, also, changes in the epigenome correlate with cancer development [29,30]. As in many other cancers, such epigenetic modifications also arise in MM [31] and can be roughly divided into modifications that occur at the DNA level and histone modifications.

DNA methylation is defined by the addition of a methyl group to the carbon-5 position of a cytosine in a cytosine-phosphate-guanine dinucleotide (CpG) and is traditionally associated with gene repression [32]. However, as many other processes in cell homeostasis, DNA methylation is not an irreversible event but, rather, a dynamic process [32,33]. 

Whole-exome analysis revealed DNA methylation modifiers, such as TET1/2/3, IDH1/2 and DNMT1/3A/B, to be frequently altered [34]. Furthermore, increases in DNMT1 and reductions in DNMT3a expression have also been reported in MM [35]. It is therefore not surprising that aberrant methylation is a hallmark of MM. MM is characterized by global hypomethylation and gene-specific hypermethylation.

#### 2.1.1. Global Hypomethylation

Importantly, changes in the global methylation pattern are associated with different stages of the disease [36]. Global hypomethylation is occurring already in the premalignant phase of monoclonal gammopathy of undetermined significance (MGUS). Hypomethylation increases during disease progression to symptomatic MM, leading to a greater genome instability via modified DNA methyltransferase (DNMT) activity, alterations in the chromatin structure, loss of imprinting and the increasing accumulation of copy number alterations [36,37]. Often, repetitive elements such as Alu, LINE-1 or SAT-α are demethylated in MM [35,38]. Interestingly, pathway analyses of the genes hypomethylated in myelomagenesis indicate an important involvement of ECM remodeling, leading to the invasive and adhesive properties of cells. This strengthens the concept that epigenetic modifications are also important in the interaction of MM plasma cells with the BMME [37]. General hypomethylation is accompanied by specific hypomethylation and the consequent gene activation in MM. Houde and colleagues described an overexpression of the NOTCH ligand JAG2 due to the hypomethylation of its promoter in cells from MGUS and MM patients but not in healthy individuals. JAG2 induces the secretion of IL-6, VEGF and IGF-1 in stromal cells, thereby showing its putative importance not only in the early development of MM but, also, in its relationship with the BMME [39].

#### 2.1.2. Gene-Specific Hypermethylation

In contrast to hypomethylation, which predominantly occurs globally in MM, hypermethylation mainly occurs on selected tumor-suppressor genes. Various studies have shown that the occurrence of specific hypermethylation is already present in the early stages of myeloma development, suggesting a role in disease progression. The hypermethylation of the proapoptotic factor DAPK; of the estrogen receptor (ER) [40]; of p15, p16, p53 and p73; of ARF and of RASSF1A [41,42], as well as the methylation of the MIR203 promoter [43] and the Wnt pathway modulators SFRP 1 and 2 [44], have all been reported. Some of these changes are already present from the stage of MGUS. The pathways affected by these genes are involved in cell death, cell cycle progression and bone homeostasis, stressing again the concept that methylation changes might influence not only the malignant potential of plasma cells but, also, their interactions with the bone and the BMME [45]. Although many studies have investigated methylation changes in MM and its precursors, in many cases, it is still unclear how these changes trigger disease progression and what impact they have on disease prognosis. Additionally, methylation changes of a specific gene might not have a causal effect *per se* but are rather a marker of more widespread changes in the whole genome. In this respect, Gonzalez-Paz and colleagues could demonstrate that the methylation of p16 had no effect on plasma cell proliferation, as well as no impact on patients’ survival. These results led the authors to postulate that the methylation of the p16 gene might serve as an overall marker for epigenetic changes rather than having a causal and prognostic effect on its own [46]. Others found that methylation and the subsequent gene silencing of p16, DAPK1, E-CAD, RBP1 and BNIP3 was associated with a poor prognosis [47,48,49]. Another gene, whose silencing through promoter hypermethylation was found to be associated with disease progression and a worse outcome, is RASSF4 [50]. De Smedt and colleagues demonstrated that RASSF4 suppression increases the pro-mitogenic activity of RAS and that the restoration of RASSF4 expression increases the effect of the MEK1/2 inhibitor trametinib, suggesting a possible strategy to more effectively target the RAS pathway in clinical practice [50].

As previously stated, methylation changes are one of the hallmarks of MM disease progression. Walker and colleagues showed that, during the progression from MGUS to MM, hypermethylation occurs in many promoters of genes involved in gene regulation (ACVR1, ARID3A, BRCA2, C19orf33, CALCA, CBX4, FOXD2, GATA4, HIPK3, HOXB8, HOXD11, ID4, IRF7, LDB1, NCOR2, ONECUT2, RAB37, RUNX2, ZIC1, ZNF385 and ZNF560) or in regulators of the cell cycle (AIF1, BCL2, CDKN2B, GAS2L1, ID4, MPHOSPH9 and PKMYT1) [36]. Other genes such as CPEB1, CD9, GJA1, BCL7, AKAP12 and BNIP have also been reported to increase their methylation status during the progression from MGUS to MM [51].

Other important genes that are often hypermethylated in MM are genes involved with Wnt signaling [44,51,52,53,54] or genes like GPX3, RBP1, SPARC and TGFBI that can modulate the myeloma development by suppressing the stimuli from the tumor microenvironment [48].

An important pathway for MM cell survival is the Jak/STAT pathway. The hypermethylation of SHP1 and SOCS-1 activates Jak/STAT, increasing MM cell survival, likely due to an increased response to cytokines like IL-6 [40,55,56].

MEG3, a gene with tumor suppression functions linked to the p53 pathway, was shown to be differentially hypermethylated in MM patients, with the highest level of methylation seen in patients with a higher tumor burden, evaluated using the Durie and Salmon Staging System. The patient population enrolled in this study was, however, very small (only 21 patients), so that no definitive conclusion on the role of MEG3 in tumor pathogenesis can be drawn [57].

Overall, the more frequently hypermethylated genes in MM are PTGS2 (100%), SFN (100%), CDKN2B (90.2%), CDH1 (88.2%), ESR1 (72.5%), HIC1 (70.5%), CCND2 (62.7%), DCC (45.1%) and TGFβR2 (39.2%), whereas RARβ (16.6%), MGMT (12.5%), AIM1 (12.5%), CDKN2A (8.3%), SOCS-1 (8.3%), CCNA1 (8.3%) and THBS1 (4.1%) are rarely found to be hypermethylated [58].

Moreover, methylation changes are involved in the progression from MM to the end-stage disease of plasma cell leukemia (PCL). Interestingly, in this case, the driving factor is not the changes in the hypomethylation pattern but in the hypermethylation of the cytokine-cytokine receptor interaction genes, Janus kinase/signal transducers and activators of transcription signaling pathways and the genes especially involved in cell–cell signaling, cell development, cell differentiation and cell adhesion. These genes are the same genes that are hypomethylated in the transition from MGUS to MM, leading to a so-called genome re-methylation at the time of disease progression [36]. Not only methylation changes in genes but, also, in noncoding RNAs such as microRNAs or long noncoding RNAs (lncRNAs) play a crucial role in MM pathogenesis. The promoter methylation of different miRNAs, usually functioning as tumor suppressors such as miR-155, miR-152, miR-10b-5p, miR-34c-3p, miR-194, miR-192 and miR-215, have been associated with oncogenic properties such as the inhibition of apoptosis and induction of proliferation. These effects are caused by the loss of the inhibitory effects of those miRNAs on the known oncogenes like DNMT1, E2F3, BTRC and MYCB or the loss of their activating effect on the known tumor suppressors such as the p53/MDM2 axis [59,60,61]. Pichiorri and colleagues reported that miR-194, miR-192 and miR-215, TP53-inducible miRNAs, were downregulated in a subset of newly diagnosed MM patients. Subsequent in vitro experiments using MM cell lines showed that the downregulation of these miRNAs is due to the hypermethylation of their promoter regions and that their re-expression was able to enhance the cell sensitivity to TP53 and to block the MM invasion and migration in vivo and in vitro [60].

Beside miRNAs, lncRNAs also play a crucial role in MM pathogenesis by regulating gene expression via epigenetic modifications. A vast variety of lncRNAs have been identified to be aberrantly expressed in MM. The epigenetic rewiring of lncRNAs in MM occurs either by further epigenetic activation of already partly activated loci or by *de novo* epigenetic activation. One example of the latter is SMILO (specific myeloma intergenic long noncoding RNA), which, due to loss of DNA methylation, becomes epigenetically activated. The activation of SMILO results in its overexpression and promotes MM cell survival via the changing expression of the genes involved in nucleosome assembly, nonsense-mediated decay, chromatin silencing and cell adhesion [62]. Overall, SMILO overexpression in MM has antiapoptotic and pro-proliferative effects due to the suppression of several interferon-stimulated genes (ISGs-ISG15, IFI27 and MX1). Targeting SMILO directly or interfering with molecules of the interferon pathway such as IFNα has shown to reduce proliferation and induce apoptosis in MM cells and could be a promising strategy to treat MM. Not only the epigenetic activation but, also, the epigenetic silencing of lncRNAs plays an important role in MM pathogenesis. It has been shown that the tumor-suppressive lncRNA BM742401 is silenced via methylation of its promoter in MM, thus interfering with plasma cell homing, metastasis, and disease progression. Interestingly, the silencing of BM742401 shows an adverse effect on the overall survival in MM patients [63]. LncRNA might also be potential targets for novel therapeutic options. For example, the oncogenic lncRNA MALAT has been proposed to regulate the proteasome machinery in MM. Targeting MALAT1 using the LNA-gapmeR antisense oligonucleotide induces anti-multiple myeloma activity, inhibiting proliferation and inducing apoptosis [64]. The therapeutic effect of LNA-gapmeR is regulated via MALAT1 and EZH2 cooperation, which upregulates KEAP1. KEAP1 is a negative regulator of NRF1 and NRF2, and NRF1/NRF2 downregulation leads to a reduced expression of antioxidant genes and increased ROS levels, triggering anti-MM activity [64].

### 2.2. Histone Modifications

In addition to changes in the DNA methylation patterns, alterations in the epigenetic regulators such as histone acetyltransferases (HATs), histone methyltransferases (HMTs) and lysine demethylases (KDMs) also contribute to disease progression. For example, in a large study on more than 400 patients, Pawlyn and colleagues identified mutations in the genes coding for the histone 1 linker protein HIST1H1E, the histone acetyltransferase (HAT) EP300 and chromatin remodelers like CHD4, ARID1A and ARID2 [34].

#### 2.2.1. Histone Methyltransferases (HMTs) in MM

The most studied epigenetic modifier in MM is the histone methyltransferase MMSET. MMSET is linked to the translocation t(4;14), which occurs in about 20% of patients with MM and is associated with a poor prognosis [64,65,66]. MMSET overexpression increases H3K36 di- and trimethylation (H3K36me2/3), decreases H3K27me2/3 and increases H3K27 acetylation (H3K27ac), thereby promoting cell proliferation, survival and the development of resistance to chemotherapeutic agents. Additionally, MMSET overexpression increases DNA repair through the recruitment of DNA damage response (DDR) to a double-strand break (DSB), thus further fostering treatment resistance [67,68]. Another study showed that MMSET contributes to myelomagenesis via increased H3K27me3 through the recruitment of EZH2 at specific loci [69]. MMSET promotes cell proliferation and survival by functioning as a coactivator of NF-κB, binding to IRF4 and increasing the expression of IRF4 [70]. Additionally, MMSET methylates AURKA on lysin 14 and 117 (K14 and K117). The methylation of K14 and K117 activates the kinase through an induction of self-phosphorylation, leading to the polyubiquitination of p53, p53 degradation and an increase in the oncogenic potential of MM cells [71]. Another important mechanism of action of MMSET is through the modulation of c-MYC via the repression of miR-126 due to an enrichment of H3K9me3 and reduction in H3ac [72]. Furthermore, H3K4 and H4K20 are also MMSET substrates [73,74]. The methylation of H3K4 and H4K20 via MMSET promotes MM cell survival [73,74]. Based on these data, Marango and colleagues postulated the role of MMSET as a transcriptional corepressor in a complex with HDAC1 and 2, mSin3a and lysine-specific demethylase 1 (LSD1/KDM1A) [73]. The methylation of H4K20 is also linked to DNA repair via the recruitment of p53-binding proteins [74]. Myeloma cells with a high MMSET expression are able to repair chemotherapy-induced DNA damage faster, and MMSET silencing was shown to increase melphalan sensitivity in vivo [67]. Furthermore, the overexpression of MMSET increases the expression of genes promoting cell cycle progression, such as CCND2, CCNG1, BRCA1, GAS1, LOH11CR2A and CHECK1; cell proliferation, such as MYBL1, LIFR and PBX1; cell adhesion, such as ADAM9 and DSG2; chromatin structure and DNA packaging, such as HMGB1, SATB1, HIST3H2A, HIST1H3A, HIST1H4A and WHSC1 [75]. MMSET overexpression also induces an increase in the activation mark H3K36me2. In MM cells harboring a t(4;14) translocation, the normal distribution of H3K36me2 is obliterated, creating a favorable transcription profile for myelomagenesis [76].

The other methyltransferases, whose expression has been shown to be increased in MM, are the histone methyltransferase PHF19 [77] and PRMT5 [78].

HMT dysfunction, reducing the H3K27me3 levels, can also lead to the overexpression of HOXA9 in MM [79]. HOX genes are involved in hematopoiesis and are suggested to have a role in leukemogenesis and myeloma development. Interestingly, although Chapman et al. reported an overexpression of HOXA9 both in MM cell lines and in primary patient samples [79], Garcia-Gomez and colleagues demonstrated an increase of HOXA4 but a decrease in HOXA9 in mesenchymal stromal cells derived from MM patients [80]. These data suggest that methylation patterns do differ and can have different pathogenic implications in the different cells involved in MM disease development and progression.

Another methyltransferase known to be important for MM disease progression and prognosis is EZH2. EZH2 is a subunit of polycomb repressor complex 2 (PRC2). Its main substrate is H3K27 [81]. EZH2 is overexpressed in MM and influences cell growth via deregulation of the cell cycle control, stimulation of IL-6R and activation of c-MYC [82,83,84]. The dual inhibition of EZH2 (acting on H3K27) and G9 (acting on H3K9) has been shown to effectively repress MM cell proliferation in vitro via the induction of cell cycle arrest and apoptosis and to repress xenograft formation in vivo. The inhibition of EZH2 and G9 reduces methylation at H3K27/H3K9, which, in turn, upregulates genes associated with the interferon and immune responses (such as OAS3, IFI6, IRF9, IFIT1 and ISG15) and suppresses genes important for MM survival, such as IRF4, MYC, KLF2 and PRDM1 [85].

DOT1L, a methyltransferase responsible for the methylation of H3K79, was also found to be crucial for the survival of myeloma cells. Higher expression levels of DOT1L were detected, especially in the early stages of multiple myeloma, MGUS and smoldering multiple myeloma (SMM). The inhibition of DOT1L was able to block the proliferation of myeloma cells in in vitro models [86,87].

#### 2.2.2. Lysine Demethylases (KDMs) in MM

Histone (H) demethylation is usually due to the demethylation of lysine (K) residues via lysine demethylases (KDMs). The reduced expression of the H3K4 demethylase KDM1A due to a germline mutation in MGUS and MM was shown to drive proliferation via MYC activation. Interestingly, transcriptomes from patients with KDM1A mutations showed enrichment in the pathways associated with both intrinsic MM pathogenesis and MM-BMME interactions in comparison with KDM1A wild-type patients, again suggesting the importance of epigenetic modifications in the interaction between MM plasma cells and the BMME [88].

Opposite to what was seen with KDM1A, another member of the lysine-specific demethylases, KDM6B, was found to be overexpressed in MM. KDM6B is a demethylase of H3K27 and is regulated by NF-kB signaling [89]. KDM6B knockdown was shown to abrogate MM cell growth and survival, whilst TNF-α and culture media previously conditioned with bone marrow stromal cells (BMSC) were able to induce KDM6B. Interestingly, the pro-proliferative effect of KDM6B in MM cells is independent of its demethylase activity, suggesting that the role of demethylases in MM might be independent of histone modifications [89]. Controversially, the loss of another H3K27 demethylase, KDM6A, was found to be associated with MM cell proliferation, clonogeneity, adhesion and tumorigenicity. KDM6A mutant cells showed decreased levels of IRF4 and c-MYC and were more sensitive to the inhibition of the histone methyltransferase EZH2 in vitro, suggesting a potential therapeutic role of EZH2 inhibitors in KDM6A-mutated MM [90].

The other lysin demethylases known to be overexpressed in MM are KDM3A, a demethylase of H3K9, and KDM5B, a demethylase of H3K4 [91,92].

H3K4 and H3K27 seem to play a pivotal role in MM. The activating mark H3K4me3 and the repressing polycomb chromatin mark H3K27me3 are enriched in MM [93], and especially, H3K27me3 is associated with the under-expression of PRC2 target genes (CXCL12, GATA2, CDH6, CIITA and ICSBP/IRF8) in most cases of MM, thereby influencing cell growth [94].

#### 2.2.3. Histone Acetyltransferases (HATs) in MM

Although alterations involving histone methylation and lysin demethylation are more prominent in MM, mutations and alterations involving HAT have also been reported. In an analysis on more than 1000 MM patients, Walker and colleagues identified, among others, mutations on the CREB-binding protein (CREBBP) and on p300 [95]. CREBBP has an intrinsic histone acetyltransferase activity, able to acetylate both histone and nonhistone proteins. p300 is another HAT that shares regions with very high sequence similarities with CREBBP. p300 interacts with phosphorylated CREB, mediating cAMP gene regulation [96]. Both CREB and p300 have been implicated in the development of hematologic cancers [97].

Acetylated histones can be recognized by proteins containing a bromodomain (BRD) [98]. These BRD-containing proteins initiate the recruitment of transcriptional activators, positively regulating gene expressions [99]. One of these activators, NSD3, has been shown to act on the chromatin microenvironment at BRD4 target genes, thereby altering the gene transcription and favoring MM pathogenesis [100]. The specific targeting of BRD-containing proteins has an antimyeloma effect. In particular, JQ1, a small inhibitor of acetyl-lysine recognition motifs, has been found to competitively bind to bromodomains [101]. JQ1 exerts its action by competing over the binding to the acetyl-lysine recognition motif, thereby displacing the bromodomains from chromatin [102]. The specific inhibition of BRD4 using JQ1 induces the downregulation of MYC transcription and, subsequently, of MYC-dependent target genes, ultimately inducing cell cycle arrest and cellular senescence in MM [103]. Targeting bromodomains to inhibit the MYC transcriptional machinery could be a way to counteract MM progression [104].

### 2.3. Epigenetic Changes and Prognosis

Global methylation changes enable the differentiation between nonmalignant and malignant cells, thereby linking methylation to the clinical outcome [36,49,105]. It has been shown that LINE-1 hypomethylation is associated with a poorer prognosis, presumably via frequent copy number losses [38]. Aoki and colleagues showed that the level of LINE-1 methylation was strongly associated with genomic breaks and with the degree of copy number losses. In analogy with what is seen in other cancers [106,107], these results made the authors postulate that LINE-1 might have a greater potential to induce genomic alterations compared to the other repetitive elements [38]. The methylation of the SOC3 gene is associated with a shortened OS, likely due to its positive effect on MM cell survival [108]. Another important prognostic factor for survival is the hypermethylation of CD9, whose downregulation outplays the immune system by making the cells less susceptible to the effects of natural killer (NK) cells [109]. Other studies have linked the hypermethylation of p16, DAPK and RARb to a more aggressive disease phenotype, a poor prognosis and a shorter OS. Patients with DCC and TGFbR2 hypermethylation have poor outcomes, bringing the authors to suggest the use of TGFbR2 hypermethylation as a prognostic factor for a reduced survival [58].

Several epigenetic modifiers have been linked to disease prognosis. Patients presenting with the translocation t(4;14), which causes the aberrant expression of FGFR3 and MMSET, have a significantly worse prognosis [75]. EZH2 overexpression has also been associated with a reduced PFS and OS [83]. In contrast with MMSET, whose poor prognostic effect can be partly overcome by treatment with proteasome inhibitors [110], the negative prognostic effect of EZH2 seems to be independent from the treatment received [111]. 

Aberrations in genes such as the KDMs KDM5B and KDM6A; PRMT5; DNA methylation modifiers like TET1/2/3; IDH1/2 and DNMT1/3A/B have all been shown to correlate with a poor prognosis and shorter OS [79,83,93]. The overexpression of histone deacetylases (HDAC) of class I, such as HDAC1, have also been correlated with a poor prognosis [112].

**Figure 1:**
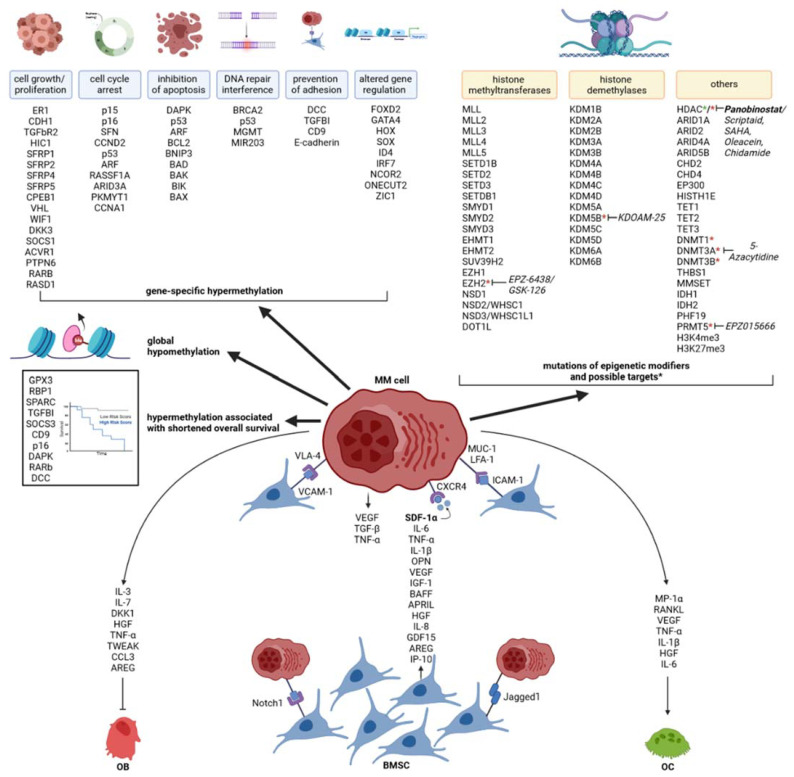
Schematic representation of the most-known epigenetic aberrations in MM and their interplay with the bone marrow microenvironment. The DNA methylation changes are categorized according to the main cellular pathway that they dysregulate. The epigenetic modifiers reported to be mutated and hypermethylated genes associated with shortened survival are indicated separately. * Identifies potential pharmacological targets. Approved compounds are signaled in bold. MM cell: multiple myeloma plasma cells, OC: osteoclasts, OB: osteoblasts and BMSC: bone marrow stromal cells.

The major epigenetic modifications occurring in MM, as well as their prognostic effect and druggability, is summarized in Figure 1.

## 3. The Role of Epigenetic Modifications in the Bone Marrow Microenvironment (BMME) and Their Role in the Development of Microenvironment-Related Drug Resistance

The most-studied epigenetic alterations in the BMME refer to MM-related symptoms, mainly bone disease. The bone homeostasis of MM patients has been widely studied, and it is clear that, among others, it is influenced by epigenetic regulators [113]. A recent study has shown that BMSCs from MM patients in different disease stages show different methylation patterns, identifying patients with SMM as those with the largest number of altered CpGs in comparison to healthy donors. Similarly, BMSC derived from patients with MGUS have the highest number of differentially variable CpG positions, followed by patients with SMM and MM, suggesting that these methylation changes affect not only the neoplastic population but, also, the BMME and might be essential for disease progression. Interestingly, coculture with MM cells was able to change the methylation profiles of BMSC derived from healthy donors to one resembling the methylation profile of MM patients. The genes mainly affected by these methylation changes are genes important for bone homeostasis, such as RUNX2 and NRP2 (hypermethylated) or SFRP2 and NFATC2 (hypomethylated). Furthermore, myeloma-induced methylation changes lead to a differential expression of numerous Homeobox genes in mesenchymal stromal cells due to an increase (HOXA9, ACVR2A and EBF2) or decrease (HOXA2, HOXA3 and HOXC5) in DNA methylation. The dual targeting of DNMTs and of the histone methyltransferase G9a was able to revert the expression of hypermethylated osteogenic regulators and prevent tumor-associated bone loss, as well as reduce the tumor burden, in a murine model [81].

The importance of RUNX2 in MM bone disease was also confirmed by others. Adamik and colleagues could show that the binding of GFI1 to the *Runx2* promoter in BMSCs initiates the recruitment of HDAC1 and EZH2, thereby enhancing the level of the *Runx2* repressive chromatin mark H3K27me3, ultimately preventing osteoblast differentiation and promoting MM bone disease. This observed effect on osteoblast differentiation could be reversed by inhibiting the activity of the epigenetic modifiers EZH2 and HDAC1 [114]. The prevention of deacetylation of the *Runx2* chromatin promoter improves myeloma osteogenesis in vitro and in vivo [115,116].

Data on the effects of epigenetic modifications in the development of bone marrow-related treatment resistance are rare. As stated, methylation in MM has been linked to ECM remodeling [37,44], suggesting a role of epigenetic modification not only in disease progression and the development of MM-related symptoms but, also, in the development of drug resistance. 

Additionally, epigenetic modifications are important in MM cell homing and adhesion to the bone marrow. Ohughi and colleagues were able to show that KDM3A overexpression interferes with MM cells homing in the bone marrow and survival via the demethylation of H3K9 and subsequent activation of KLF2 and IRF4 [92].

The development of drug resistance can be triggered by the BMME via the modification of epigenetic markers such as H3K27. Kikuchi and colleagues showed that MM cell adhesion with the BMME induces the phosphorylation of EZH2. Phosphorylated EZH2 is inactive, and its inactivation reverses drug-induced hypermethylation at H3K27. The demethylation of H3K27 leads to the activation of antiapoptotic genes like IGF1, BCL2 and HIF1α and restores CAM-DR. These data suggest that epigenetic drugs inhibiting the IGF-1R/PI3K/Akt pathway might be promising agents to overcome the treatment resistance by promoting EZH2 dephosphorylation and H3K27 hypermethylation [117]. The BMME can also initiate drug resistance by regulating miRNAs. The stroma-mediated downregulation of miR-101-3p and consequent upregulation of survivin has been shown to protect MM cells against antimyeloma drugs [118]. Targeting the miR-101-3p/survivin axis in MM by either the overexpression of miR-101-3p or by the silencing of survivin induces apoptosis even in the presence of BMSCs, thus overcoming the microenvironment-induced drug resistance [118].

The targeting of epigenetic modifiers can be beneficial in MM, as they interfere with proliferation and apoptosis. Coculture of MM cells with BMSCs induces the expression of HDAC3 in the latter, leading to an increased MM cell proliferation. Ho and colleagues showed that the knockdown of HDAC3 inhibits IL-6 trans-signaling, decreasing MM cell proliferation. Furthermore, HDAC3 knockdown leads to a change in the exosome quantity and quality, downregulating the pro-survival of miR380, -383, -15b, -9986 and -5191 and inducing cell growth arrest [119].

These data are in line with the evidence showing that a treatment with epi-drugs can overcome bone marrow microenvironment-mediated drug resistance [120]. Different studies have shown that HDAC inhibitors can downregulate the soluble factors important for SM-DR, such as IGF-1, IGF-1R and IL-6 [121,122]. HDAC inhibition was shown to reduce not only SM-DR but, also, CAM-DR. Preclinical data indicate that cocultures with BMSCs were not able to revert apoptosis induced by HDAC inhibition, suggesting that epigenetic treatments might be able to overcome the protective effect of the BMME on MM cells. 

Not only HDACs but, also, HATs are important in the relationship between the BMME and MM cells. Loss of the functional HAT CREB has been shown to be implicated in the disruption of the hematopoietic microenvironment [123], suggesting that modifications in HAT might play a relevant role in the interactions between MM cells and their normal counterparts.

Therefore, epigenetic interventions can not only be active on MM cells themselves but, in addition, can exert a beneficial impact by overcoming the protective influence of the BMME on MM cell survival, proliferation and chemoresistance.

## 4. Therapeutic Strategies Addressing Aberrant Epigenetics in Multiple Myeloma

Epigenetic modifications are reversible and, therefore, display a promising target for cancer treatment [124]. Various drugs have been designed that target enzymes to reverse aberrant epigenetics. Some of these treatments have proven to be beneficial in various types of cancers and are part of the common clinical practice in some hematological malignancies, such as myelodysplastic syndrome and acute myeloid leukemia [29,124]. Similarly to what is seen in myeloid malignancies, a lot of research has been done to identify possible targets in the epigenome that display promising effects against MM plasma cells.

Despite very active preclinical research, so far, epigenetic treatments have shown limited efficacy in MM. To date, the only epigenetic drug approved for MM treatment is the pan-HDAC inhibitor panobinostat. Panobinostat acts, among others, on HDAC6 and, therefore, interferes with the chaperone function of HSP90, leading to the degradation of PPP3CA and, subsequently, reducing the cell growth [125]. Panobinostat showed moderate single agent activity [126] but can act synergistically with proteasome inhibitors [127,128] and was approved in 2015 by the FDA [129] and EMA [130] in combination with bortezomib and dexamethasone based on the data of the phase III PANORAMA-1 trial [131].

The PANORAMA-1 trial was a multicenter, randomized, placebo-controlled, double-blinded phase III trial of patients with relapsed or refractory MM, who had received one to three prior lines of treatment. The trial included 768 patients that were randomly assigned to bortezomib and dexamethasone in combination with panobinostat or placebo. Patients that were refractory to bortezomib were excluded. Overall, patients receiving panobinostat showed a better outcome compared to patients in the placebo arm, with a higher rate of high-quality responses (at least a near-complete response 27.6% vs. 15.7% for panobinostat and placebo, respectively, *p* = 0.00006) and a longer PFS (12 vs. 8.1 months for panobinostat and placebo, respectively, *p* < 0.0001) [131]. The benefit of panobinostat was more evident in patients that had received prior treatment with both bortezomib and an IMiD (median PFS 10.6 vs. 5.8 months for panobinostat and the placebo, respectively, *p* = 0.0011) [132]. Unfortunately, panobinostat did not significantly improve the overall survival [133]. Additionally, the treatment with panobinostat was affected by a relatively high toxicity. In the PANORAMA-1 trial, 36% of patients receiving panobinostat were not able to complete the study due to adverse events, compared with only 17% in the placebo arm. The major adverse events of panobinostat, associated with its nonspecific mode of action, are gastrointestinal events such as diarrhea and cardiovascular events such as the occurrence of arrhythmias [134]. To improve its tolerability, the ongoing PANORAMA-3 trial is currently investigating reduced doses of panobinostat in combination with subcutaneous bortezomib and dexamethasone (NCT02654490). The initial results of the trial show an improved tolerability with the subcutaneous administration of bortezomib. Despite the responses being higher in patients treated with 20 mg of panobinostat, the best-tolerated schedule is the administration of panobinostat 10 mg three times a week [135]. Other studies planned to evaluate combinations of panobinostat with the IMiD lenalidomide [136] or with the second-generation PI carfilzomib [137]. The majority of these studies were discontinued due to poor accrual. The clinical trials involving panobinostat are summarized in Table 1.

Beside the approved panobinostat, other HDAC inhibitors have been evaluated in multiple myeloma. Based on promising in vitro activity alone and in combination [122,143,144,145], vorinostat, a pan-HDAC inhibitor approved for the treatment of cutaneous T-cell lymphoma, was also studied in MM patients. Studies on vorinostat have included combination therapy with bortezomib [146,147] in relapsed and refractory patients; combinations with bortezomib, lenalidomide and dexamethasone in newly diagnosed MM patients [138] and maintenance treatment in combinations with lenalidomide [148] or bortezomib [149]. Overall, despite showing some degree of responses, with ORR ranging from 96% to 56% according to the different population of patients included in the studies, these trials confirmed an increased toxicity of pan-HDAC inhibitors, and vorinostat has, so far, not been approved for MM treatment.

The same problem of arising toxicities and insufficient effects when applied as a monotherapy was observed in other clinical trials of HDAC inhibitors in relapsed MM patients. For example, the HDAC inhibitor romidepsin has been investigated in a phase II trial, where it was reported to induce some biological effects such as the stabilization of M-protein production or the resolution of hypercalcemia and improvement of bone pain; yet, no significant benefit was observed, as it did not induce tumor regression [150]. The HDAC inhibitor ITF2357 also failed to prevent disease progression and, in addition, induced severe adverse effects like thrombocytopenia, neutropenia and pneumonia, as well as gastrointestinal toxicities and cardiovascular events [151].

To reduce HDAC treatment toxicities, attempts have been made to develop more specific HDAC inhibitors, such as the HDAC6 inhibitor ricolinostat (ACY-1215) [152]. Ricolinostat showed no single agent activity but was active in combination with bortezomib. Nevertheless, despite the more selective HDAC inhibition compared to panobinostat and vorinostat, gastrointestinal toxicity remains a clinically relevant problem. Another limiting aspect of the clinical development of ricolinostat is the challenge in deriving a solid dose formulation and the observed exposure plateau [153].

The compound ACY-241 is very similar in structure to ricolinostat, without showing the same exposure plateau, and is also available as an oral formulation. ACY-241 is currently being tested in a phase I trial in combination with pomalidomide and dexamethasone (NCT02400242) [154].

A452, another selective HDAC6 inhibitor, has shown in vitro efficacy in combination with IMiDs and dexamethasone [155].

Not only HDAC inhibitors but, also, other targets are being investigated as potential therapeutic strategies to treat MM. The perhaps most-promising target identified is the oncogene EZH2. The inhibition of EZH2 has been shown to decrease the levels of H3K27 trimethylation (H3K27me3), an effect that was potentiated by the concomitant loss of KDM6 [91]. The reduction of H3K27me3 can induce cell death and apoptosis. H3K27me3 is essential for the suppression of miR-29b. If H3K27me3 is inhibited, miR-29b is upregulated, leading to a decrease in the levels of pro-survival proteins like SP1, MCL-1 and CDK6 [94,156]. The upregulation of miR-29b is not the only mode of action of EZH2 inhibition. The downregulation of oncogenes such as IRF-4, XBP-1, PRDM1/BLIMP-1 and c-MYC, as well as the upregulation of tumor-suppressive microRNAs like miR-125a-3p and miR-320c, have also been shown [85].

Other promising targets, whose synergistic inhibition shows antimyeloma effects, are DNMT1 and HDAC3, due to downregulation of c-MYC [157]. Another potential target is PRMT5, as its inhibition was shown to reduce MM cell survival via the p53 axis [79].

Targeting the epigenetic aberrations in MM is able to reduce MM cell survival in vitro. One example is the polyphenol Oleacein, which has HDAC inhibitory properties and leads to cell cycle arrest and apoptosis via caspase-8 activation and the downregulation of Sp1 [158]. Another compound that reduces MM cell viability is Scriptaid, which induces cell cycle arrest at the G2/M phase and apoptosis via p21 regulation through the alteration of H3 acetylation [159]. The inhibition of KDM5B using the selective inhibitor KDOAM-25 leads to an enrichment of H3K4 methylation, inducing cell cycle arrest and preventing cell proliferation [93].

Interestingly, some compounds do not only show single agent activity but are able to reverse drug resistances in vitro. This phenomenon is very important, as MM is still an incurable disease, and drug resistances is a common and frequent event in MM patients. The occurrence of drug resistance in MM is linked, among others, to epigenetic dysregulation. For example, chemotherapeutic agents like topotecan, doxorubicin and VP-16 trigger drug resistance via an increase in ABCG2 expression via promoter demethylation [160]. Another study reported a connection between IMiDs resistance and epigenetic alterations at the chromatin and DNA levels, which could be restored using EZH2 inhibitors in combination with targeting DNA methylation [161]. Sensitization to the histone deacetylase (HDAC) inhibitor panobinostat could be achieved by pretreatment with the EZH2 inhibitors EPZ-6438 and GSK-126 in a synergistic manner [162]. GSK-126 is believed to act through the involvement of the mitochondrial pathway via MCL-1 cleavage of caspase-3 and induction of apoptosis [163]. One other compound with a sensitizing effect is the HDAC inhibitor chidamide, which reverses resistance to the PI bortezomib. Chidamide inhibits type I HDACs, thereby promoting H3 and H4 acetylation. The acetylation of H3 and H4 leads to a reduction in the expression levels of cyclin D1 and c-MYC and enrichment of the expression levels of p53 and p21, with a consequent cell cycle arrest from G0 to G1. Additionally, chidamide can induce apoptosis through interference in the Bax/BCL-2 ratio [164]. The hypoxia-selective epigenetic agent RRx-001, which has shown in vivo antimyeloma effects in mouse models, can sensitize in vitro cells to conventional therapies such as bortezomib, pomalidomide and the HDAC inhibitor SAHA via the downregulation of DNMTs and subsequent inhibition of DNA methylation [165]. The DNA methyltransferase inhibitor 5-azacytidine, functioning via DNA repair and proapoptotic pathways, is able to re-sensitize MM cells to doxorubicin [166]. The 5-azacytidine derivate 5-aza-2-deoxycytidine (decitabine) is able to restore in vitro the function of tumor-suppressor genes [48] and can revert the methylation-induced inactivation of RASD1, thus overcoming dexamethasone resistance [167].

## 5. Conclusions and Future Perspective

Although research has revealed various dysregulations of the epigenome in MM, only one epigenetic treatment has been approved for this disease so far. Yet, the preclinical data summarized in this review clearly show that epigenetic treatments might be beneficial in reducing the cell viability, in interfering with the protective effect of the BMME and in sensitizing cells to conventionally applied medications. Importantly, epigenetic modifiers, such as HDACs or DNMTs, alter not only histones and DNA but might also affect the post-transcriptional modifications (PTMs) of other proteins. For example, HDAC inhibitors have been shown to disrupt proteostasis by targeting the unfolded protein response (UPR) pathway [168] and may alter other proteins important for MM pathogenesis, such as p53, Hsp90 and the aggresome [169]. Extremely intriguing is the increasing evidence that epigenetic dysregulation affects not only MM cells, but, also, the BMME, being responsible for disease progression and the development of MM-associated events such as osteolysis and the acquisition of treatment resistance. Whether the so-called “off-target effects” of epigenome regulators also play a role in the interactions between the plasma cells and the microenvironment has yet to be demonstrated, but is plausible. Interfering with the epigenome to inhibit the interactions between malignant plasma cells and the bone marrow microenvironment might still be a promising strategy in the future. When the aim is not an effect on the MM cells but, rather, a re-sensitizing approach, the use of low doses of epigenetic drugs in combination with known anti-MM treatments might be able to overcome the microenvironment-induced treatment resistance and reduce MM-related events with acceptable toxicities. Additional research in this field is warranted, as only with a better understanding of MM biology and of the complex interplay within the BMME we can hope to further improve the treatments of this still incurable disease.

## Figures and Tables

**Table 1:** The main trials with panobinostat in multiple myeloma.

Treatment	Phase	Number of Patients	Patient Cohorts	Median Follow-Up	Results			Other Information	Reference	Trial ID
					ORR	Survival				
						PFS	OS			
panobinostat + bortezomib	Ib	47 (dose escalation) 15 (dose expansion)	RRMM	NR	73.3% (expansion phase); 52.9% (escalation phase); among bortezomib- refractory patients: 26.3%	NA	NA	42.1% of patients reached MR or better	[131]	NCT00532389
panobinostat + carfilzomib	I	32	RRMM	27 months	ORR in all patients 57%, ORR among patients treated with MTD 63%, no differences between bortezomib- refractory and bortezomib- sensitive patients (57% vs. 55%, *p* = 1)	8 months (95% CI: 5–11 months)	23 months (95% CI: 16-NA months)	CBF 68%; no differences between bortezomib refractory and sensitive patients in median PFS and OS	[138]	NCT01549431
panobinostat + lenalidomide + bortezomib + dexamethasone	I	55 (45 evaluable patients treated at MTD)	NDMM	for all patients: 40 months (95% CI: 6.3–61.7 months), for patients treated with MTD: 39 months (95% CI: 6.3–61.7 months)	ORR among patients treated with MTD: 96%	not reached	not reached		[139]	NCT01440582
panobinostat + bortezomib	I	NR	RRMM					terminated (study drug unavailable) no results available		NCT00891033
panobinostat + lenalidomide + dexamethasone	I	46	RRMM					no results available		NCT00532675
panobinostat + everolimus	I	11	RRMM					no results available		NCT00962507
panobinostat + Melphalan	I/II	40	RRMM	NA	7.5%	TTP: 1.6 months (95% CI: 0.6–2.7)	not reached		[140]	NCT00743288
panobinostat + lenalidomide + carfilzomib + dexamethasone	I/II	NR	NDMM					withdrawn study drug unavailable no results available		NCT0280216
panobinostat + ixazomib (DL1: 3 mg; DL2: 4 mg) + dexamethasone	I/II	16	RRMM	NA	NA	DL1: 1.2 months (95% CI 0.7–6) DL2: 3.5 months (95% CI 0.9–7.4)	DL1: 12.8 months (95% CI 1.7–30.4) DL2: 17.6 months (95% CI 11.9–22.9)			NCT02057640
panobinostat + everolimus	I/II	124	RRMM or lymphoma					no results available		NCT00918333
panobinostat + carfilzomib	I/II	66	RRMM	26.1 months (95% CI: 0–72.5 months)	84.4%	10.3 months (95% CI 6.1–13.9)	44.6 months (95% CI: 20.8–NA months)	CBF: 90.6%	[137]	NCT01496118
panobinostat + lenalidomide + dexamethasone	II	27	RRMM	NA	40.7% (36.4% in lenalidomide refractory patients)	7.1 months for all patients (6.5 months for refractory patients)	not reached	CBF: 74%, DCR: 96%	[136]	NCT01651039
panobinostat	II	38	RRMM					terminated no results available		NCT00445068
panobinostat + bortezomib + dexamethasone (PANORAMA-2)	II	55	RRMM	8.3 months	34.5% (1 nCR, 18 PR, 10 MR)	5.4 months (95% CI 2.6–6.7 months)	not reached after median follow-up of 8.3 months		[141]	NCT0108360
panobinostat + lenalidomide + bortezomib + dexamethasone (PANORAMA-4)	II	6	new diagnose					terminated (due to low enrollment)		NCT02720510
panobinostat + carfilzomib + dexamethasone (PANORAMA-5)	II	0	RRMM					withdrawn		NCT02756663
panobinostat + bortezomib + dexamethasone	II	31	RRMM	NA	80.6% (95% CI 62.5–92.5 months)	15.3 months (95% CI 10.4–31.4 months)	not estimable	CR + nCR 48.4% (90% CI: 33.6–63.2)	[142]	NCT02290431
panobinostat + bortezomib + dexamethasone (PANORAMA-1)	III	767	relapsed MM	NA	60.7% vs. 54.6%	11.99 m vs. 8.08 m (HR 0.63, 95% CI 0.52–0.76; *p* < 0.0001)	33.64 m vs. 30.39 m (HR 0.87, 95% CI 0.69–1.10; *p* = 0.26)	CR or nCR 27.6% vs. 15.7% (*p* = 0.00006)	[131]	NCT01023308
panobinostat	II	30	maintenance after ASCT					not yet available		NCT02722941
panobinostat + bortezomib + dexamethasone (PANORAMA-3)	II	249	RRMM, previously exposed to IMiDs	14.7 months (95% CI 7.8–24.1 months)	20-mg panobinostat Thrice-weekly: 62.2% (95% CI 50.8–72.7); 20-mg panobinostat Twice-weekly: 65.1% (53.8–75.2); 10-mg panobinostat Thrice-weekly: 50.6% (39.4–61.8)	not reached	not reached		[135]	NCT02654990
panobinostat + gemcitabine + hydrochloride + busulfan + melphalan	II	80	RRMM, before ASCT	NA	NA	NA	NA	not yet available		NCT02506959
panobinostat + carfilzomib + dexamethasone	II	9	RRMM					terminated (loss of funding)		NCT03256045

ORR: overall response rate, PFS: progression-free survival, OS: overall survival, RRMM: relapsed refractory multiple myeloma, NDMM: newly diagnosed multiple myeloma, CR: complete response, VGPR: very good partial response, PR: partial response, NA: not available, NR: not reported, ASCT: autologous stem cell transplantation, CBF: clinical benefit rate, DCR: disease control rate (SD or greater for >2 months) and TTP: time to progression.

## References

[1] Van de Donk N.W.C.J., Pawlyn C., Yong K.L. (2021). Multiple myeloma. Lancet Lond. Engl..

[2] Cavo M., Tacchetti P., Patriarca F., Petrucci M.T., Pantani L., Galli M., Di Raimondo F., Crippa C., Zamagni E., Palumbo A. (2010). Bortezomib with thalidomide plus dexamethasone compared with thalidomide plus dexamethasone as induction therapy before, and consolidation therapy after, double autologous stem-cell transplantation in newly diagnosed multiple myeloma: A randomised phase 3 study. Lancet.

[3] Moreau P., Attal M., Hulin C., Arnulf B., Belhadj K., Benboubker L., Béné M.C., Broijl A., Caillon H., Caillot D. (2019). Bortezomib, thalidomide, and dexamethasone with or without daratumumab before and after autologous stem-cell transplantation for newly diagnosed multiple myeloma (CASSIOPEIA): A randomised, open-label, phase 3 study. Lancet.

[4] Facon T., Kumar S., Plesner T., Orlowski R.Z., Moreau P., Bahlis N., Basu S., Nahi H., Hulin C., Quach H. (2019). Daratumumab plus Lenalidomide and Dexamethasone for Untreated Myeloma. N. Engl. J. Med..

[5] Dimopoulos M., Quach H., Mateos M.-V., Landgren O., Leleu X., Siegel D., Weisel K., Yang H., Klippel Z., Zahlten-Kumeli A. (2020). Carfilzomib, dexamethasone, and daratumumab versus carfilzomib and dexamethasone for patients with relapsed or refractory multiple myeloma (CANDOR): Results from a randomised, multicentre, open-label, phase 3 study. Lancet Lond. Engl..

[6] Mateos M.-V., Dimopoulos M.A., Cavo M., Suzuki K., Jakubowiak A., Knop S., Doyen C., Lucio P., Nagy Z., Kaplan P. (2018). Daratumumab plus Bortezomib, Melphalan, and Prednisone for Untreated Myeloma. N. Engl. J. Med..

[7] Dimopoulos M.A., Oriol A., Nahi H., San-Miguel J., Bahlis N.J., Usmani S.Z., Rabin N., Orlowski R.Z., Komarnicki M., Suzuki K. (2016). Daratumumab, Lenalidomide, and Dexamethasone for Multiple Myeloma. N. Engl. J. Med..

[8] Kumar S.K., Rajkumar S.V., Dispenzieri A., Lacy M.Q., Hayman S.R., Buadi F.K., Zeldenrust S.R., Dingli D., Russell S.J., Lust J.A. (2008). Improved survival in multiple myeloma and the impact of novel therapies. Blood.

[9] Kumar S.K., Dispenzieri A., Lacy M.Q., Gertz M.A., Buadi F.K., Pandey S., Kapoor P., Dingli D., Hayman S.R., Leung N. (2014). Continued improvement in survival in multiple myeloma: Changes in early mortality and outcomes in older patients. Leukemia.

[10] Tacchetti P., Pantani L., Patriarca F., Petrucci M.T., Zamagni E., Dozza L., Galli M., Di Raimondo F., Crippa C., Boccadoro M. (2020). Bortezomib, thalidomide, and dexamethasone followed by double autologous haematopoietic stem-cell transplantation for newly diagnosed multiple myeloma (GIMEMA-MMY-3006): Long-term follow-up analysis of a randomised phase 3, open-label study. Lancet Haematol..

[11] Gandhi U.H., Cornell R.F., Lakshman A., Gahvari Z.J., McGehee E., Jagosky M.H., Gupta R., Varnado W., Fiala M.A., Chhabra S. (2019). Outcomes of patients with multiple myeloma refractory to CD38-targeted monoclonal antibody therapy. Leukemia.

[12] Basak G.W., Srivastava A.S., Malhotra R., Carrier E. (2009). Multiple myeloma bone marrow niche. Curr. Pharm. Biotechnol..

[13] Romano A., Conticello C., Cavalli M., Vetro C., La Fauci A., Parrinello N.L., Di Raimondo F. (2014). Immunological dysregulation in multiple myeloma microenvironment. BioMed Res. Int..

[14] Morgan G.J., Walker B.A., Davies F.E. (2012). The genetic architecture of multiple myeloma. Nat. Rev. Cancer.

[15] Korde N., Kristinsson S.Y., Landgren O. (2011). Monoclonal gammopathy of undetermined significance (MGUS) and smoldering multiple myeloma (SMM): Novel biological insights and development of early treatment strategies. Blood.

[16] Nefedova Y., Landowski T.H., Dalton W.S. (2003). Bone marrow stromal-derived soluble factors and direct cell contact contribute to de novo drug resistance of myeloma cells by distinct mechanisms. Leukemia.

[17] Wang J., Hendrix A., Hernot S., Lemaire M., De Bruyne E., Van Valckenborgh E., Lahoutte T., De Wever O., Vanderkerken K., Menu E. (2014). Bone marrow stromal cell–derived exosomes as communicators in drug resistance in multiple myeloma cells. Blood.

[18] Roccaro A.M., Sacco A., Maiso P., Azab A.K., Tai Y.-T., Reagan M., Azab F., Flores L.M., Campigotto F., Weller E. (2013). BM mesenchymal stromal cell-derived exosomes facilitate multiple myeloma progression. J. Clin. Investig..

[19] Akiyama M., Hideshima T., Hayashi T., Tai Y.-T., Mitsiades C.S., Mitsiades N., Chauhan D., Richardson P., Munshi N.C., Anderson K.C. (2002). Cytokines Modulate Telomerase Activity in a Human Multiple Myeloma Cell Line. Cancer Res..

[20] Zipori D. (2010). The hemopoietic stem cell niche versus the microenvironment of the multiple myeloma-tumor initiating cell. Cancer Microenviron..

[21] Fowler J.A., Mundy G.R., Lwin S.T., Edwards C.M. (2012). Bone marrow stromal cells create a permissive microenvironment for myeloma development: A new stromal role for Wnt inhibitor Dkk1. Cancer Res..

[22] Di Marzo L., Desantis V., Solimando A.G., Ruggieri S., Annese T., Nico B., Fumarulo R., Vacca A., Frassanito M.A. (2016). Microenvironment drug resistance in multiple myeloma: Emerging new players. Oncotarget.

[23] Azab A., Quang P., Azab F., Pitsillides C., Thompson B., Chonghaile T., Patton J., Maiso P., Monrose V., Sacco A. (2011). P-selectin glycoprotein ligand regulates the interaction of multiple myeloma cells with the bone marrow microenvironment. Blood.

[24] De Haart S.J., Van De Donk N.W., Minnema M.C., Huang J.H., Aarts-Riemens T., Bovenschen N., Yuan H., Groen R.W.J., McMillin D.W., Jakubikova J. (2013). Accessory Cells of the Microenvironment Protect Multiple Myeloma from T-Cell Cytotoxicity through Cell Adhesion-Mediated Immune Resistance. Clin. Cancer Res..

[25] Zheng Y., Cai Z., Wang S., Zhang X., Qian J., Hong S., Li H., Wang M., Yang J., Yi Q. (2009). Macrophages are an abundant component of myeloma microenvironment and protect myeloma cells from chemotherapy drug-induced apoptosis. Blood.

[26] Fan F., Bashari M.H., Morelli E., Tonon G., Malvestiti S., Vallet S., Jarahian M., Seckinger A., Hose D., Bakiri L. (2017). The AP-1 transcription factor JunB is essential for multiple myeloma cell proliferation and drug resistance in the bone marrow microenvironment. Leukemia.

[27] Alonso S., Hernandez D., Chang Y.-T., Gocke C.B., McCray M., Varadhan R., Matsui W.H., Jones R.J., Ghiaur G. (2016). Hedgehog and retinoid signaling alters multiple myeloma microenvironment and generates bortezomib resistance. J. Clin. Investig..

[28] Furukawa Y., Kikuchi J. (2015). Molecular pathogenesis of multiple myeloma. Int. J. Clin. Oncol..

[29] Di Costanzo A., Del Gaudio N., Migliaccio A., Altucci L. (2014). Epigenetic drugs against cancer: An evolving landscape. Arch. Toxicol..

[30] Sharma S., Kelly T.K., Jones P.A. (2010). Epigenetics in cancer. Carcinogenesis.

[31] De Smedt E., Lui H., Maes K., De Veirman K., Menu E., Vanderkerken K., De Bruyne E. (2018). The Epigenome in Multiple Myeloma: Impact on Tumor Cell Plasticity and Drug Response. Front. Oncol..

[32] Smith Z.D., Meissner A. (2013). DNA methylation: Roles in mammalian development. Nat. Rev. Genet..

[33] Wu H., Zhang Y. (2014). Reversing DNA methylation: Mechanisms, genomics, and biological functions. Cell.

[34] Pawlyn C., Kaiser M.F., Heuck C., Melchor L., Wardell C.P., Murison A., Chavan S.S., Johnson D.C., Begum D.B., Dahir N.M. (2016). The Spectrum and Clinical Impact of Epigenetic Modifier Mutations in Myeloma. Clin. Cancer Res..

[35] Bollati V., Fabris S., Pegoraro V., Ronchetti D., Mosca L., Deliliers G.L., Motta V., Bertazzi P.A., Baccarelli A., Neri A. (2009). Differential repetitive DNA methylation in multiple myeloma molecular subgroups. Carcinogenesis.

[36] Walker B.A., Wardell C.P., Chiecchio L., Smith E.M., Boyd K.D., Neri A., Davies F.E., Ross F.M., Morgan G.J. (2011). Aberrant global methylation patterns affect the molecular pathogenesis and prognosis of multiple myeloma. Blood.

[37] Salhia B., Baker A., Ahmann G., Auclair D., Fonseca R., Carpten J. (2010). DNA methylation analysis determines the high frequency of genic hypomethylation and low frequency of hypermethylation events in plasma cell tumors. Cancer Res..

[38] Aoki Y., Nojima M., Suzuki H., Yasui H., Maruyama R., Yamamoto E., Ashida M., Itagaki M., Asaoku H., Ikeda H. (2012). Genomic vulnerability to LINE-1 hypomethylation is a potential determinant of the clinicogenetic features of multiple myeloma. Genome Med..

[39] Houde C., Li Y., Song L., Barton K., Zhang Q., Godwin J., Nand S., Toor A., Alkan S., Smadja N.V. (2004). Overexpression of the NOTCH ligand JAG2 in malignant plasma cells from multiple myeloma patients and cell lines. Blood.

[40] Chim C., Liang R., Leung M., Kwong Y. (2007). Aberrant gene methylation implicated in the progression of monoclonal gammopathy of undetermined significance to multiple myeloma. J. Clin. Pathol..

[41] Geraldes C., Gonçalves A.C., Cortesão E., Pereira M.I., Roque A., Paiva A., Ribeiro L., Nascimento-Costa J.M., Sarmento-Ribeiro A.B. (2016). Aberrant p15, p16, p53, and DAPK Gene Methylation in Myelomagenesis: Clinical and Prognostic Implications. Clin. Lymphoma Myeloma Leuk..

[42] Stanganelli C., Arbelbide J., Fantl D.B., Corrado C., Slavutsky I. (2010). DNA methylation analysis of tumor suppressor genes in monoclonal gammopathy of undetermined significance. Ann. Hematol..

[43] Wong K.-Y., Liang R., So C.-C., Jin D.-Y., Costello J.F., Chim C.-S. (2011). Epigenetic silencing of MIR203 in multiple myeloma. Br. J. Haematol..

[44] Jost E., Gezer D., Wilop S., Suzuki H., Herman J.G., Osieka R., Galm O. (2009). Epigenetic dysregulation of secreted Frizzled-related proteins in multiple myeloma. Cancer Lett..

[45] Kobune M., Chiba H., Kato J., Kato K., Nakamura K., Kawano Y., Takada K., Takimoto R., Takayama T., Hamada H. (2007). Wnt3/RhoA/ROCK signaling pathway is involved in adhesion-mediated drug resistance of multiple myeloma in an autocrine mechanism. Mol. Cancer Ther..

[46] Gonzalez-Paz N., Chng W.J., McClure R.F., Blood E., Oken M.M., Van Ness B., James C.D., Kurtin P.J., Henderson K., Ahmann G.J. (2007). Tumor suppressor p16 methylation in multiple myeloma: Biological and clinical implications. Blood.

[47] Wong K.Y., Chim C.S. (2015). DNA methylation of tumor suppressor protein-coding and non-coding genes in multiple myeloma. Epigenomics.

[48] Kaiser M.F., Johnson D.C., Wu P., Walker B.A., Brioli A., Mirabella F., Wardell C.P., Melchor L., Davies F.E., Morgan G.J. (2013). Global methylation analysis identifies prognostically important epigenetically inactivated tumor suppressor genes in multiple myeloma. Blood.

[49] Martínez-Baños D., Sánchez-Hernández B., Jiménez G., Barrera-Lumbreras G., Barrales-Benítez O. (2017). Global methylation and promoter-specific methylation of the P16, SOCS-1, E-cadherin, P73 and SHP-1 genes and their expression in patients with multiple myeloma during active disease and remission. Exp. Ther. Med..

[50] De Smedt E., Maes K., Verhulst S., Lui H., Kassambara A., Maes A., Robert N., Heirman C., Cakana A., Hose D. (2018). Loss of RASSF4 Expression in Multiple Myeloma Promotes RAS-Driven Malignant Progression. Cancer Res..

[51] Heller G., Schmidt W.M., Ziegler B., Holzer S., Müllauer L., Bilban M., Zielinski C.C., Drach J., Zöchbauer-Müller S. (2008). Genome-wide transcriptional response to 5-aza-2’-deoxycytidine and trichostatin a in multiple myeloma cells. Cancer Res..

[52] Heuck C.J., Mehta J., Bhagat T., Gundabolu K., Yu Y., Khan S., Chrysofakis G., Schinke C., Tariman J., Vickrey E. (2013). Myeloma is characterized by stage-specific alterations in DNA methylation that occur early during myelomagenesis. J. Immunol..

[53] Chim C.S., Pang R., Fung T.K., Choi C.L., Liang R. (2007). Epigenetic dysregulation of Wnt signaling pathway in multiple myeloma. Leukemia.

[54] Pompeia C., Hodge D.R., Plass C., Wu Y.-Z., Marquez V.E., Kelley J.A., Farrar W.L. (2004). Microarray analysis of epigenetic silencing of gene expression in the KAS-6/1 multiple myeloma cell line. Cancer Res..

[55] Galm O., Yoshikawa H., Esteller M., Osieka R., Herman J.G. (2003). SOCS-1, a negative regulator of cytokine signaling, is frequently silenced by methylation in multiple myeloma. Blood.

[56] Chim C.-S., Fung T.-K., Cheung W.-C., Liang R., Kwong Y.-L. (2004). SOCS1 and SHP1 hypermethylation in multiple myeloma: Implications for epigenetic activation of the Jak/STAT pathway. Blood.

[57] Benetatos L., Dasoula A., Hatzimichael E., Georgiou I., Syrrou M., Bourantas K.L. (2008). Promoter hypermethylation of the MEG3 (DLK1/MEG3) imprinted gene in multiple myeloma. Clin. Lymphoma Myeloma.

[58] De Carvalho F., Colleoni G.W.B., Almeida M.S.S., Carvalho A.L., Vettore A.L. (2009). *TGFβR2* aberrant methylation is a potential prognostic marker and therapeutic target in multiple myeloma. Int. J. Cancer.

[59] Krzeminski P., Sarasquete M.E., Misiewicz-Krzeminska I., Corral R., Corchete L.A., Martín A.A., García-Sanz R., San Miguel J.F., Gutiérrez N.C. (2015). Insights into epigenetic regulation of microRNA-155 expression in multiple myeloma. Biochim. Biophys. Acta.

[60] Pichiorri F., Suh S.-S., Rocci A., De Luca L., Taccioli C., Santhanam R., Zhou W., Benson D.M., Hofmainster C., Alder H. (2010). Downregulation of p53-inducible microRNAs 192, 194, and 215 impairs the p53/MDM2 autoregulatory loop in multiple myeloma development. Cancer Cell.

[61] Zhang W., Wang Y.E., Zhang Y., Leleu X., Reagan M., Zhang Y., Mishima Y., Glavey S., Manier S., Sacco A. (2014). Global epigenetic regulation of microRNAs in multiple myeloma. PLoS ONE.

[62] Carrasco-Leon A., Ezponda T., Meydan C., Valcárcel L.V., Ordoñez R., Kulis M., Garate L., Miranda E., Segura V., Guruceaga E. (2021). Characterization of complete lncRNAs transcriptome reveals the functional and clinical impact of lncRNAs in multiple myeloma. Leukemia.

[63] Li Z., Kumar S., Jin D.-Y., Calin G.A., Chng W.-J., Siu K.-L., Poon M.-W., Chim C.S. (2020). Epigenetic silencing of long non-coding RNA BM742401 in multiple myeloma: Impact on prognosis and myeloma dissemination. Cancer Cell Int..

[64] Amodio N., Stamato M.A., Juli G., Morelli E., Fulciniti M., Manzoni M., Taiana E., Agnelli L., Cantafio M.E.G., Romeo E. (2018). Drugging the lncRNA MALAT1 via LNA gapmeR ASO inhibits gene expression of proteasome subunits and triggers anti-multiple myeloma activity. Leukemia.

[65] Avet-Loiseau H., Attal M., Moreau P., Charbonnel C., Garban F., Hulin C., Leyvraz S., Michallet M., Yakoub-Agha I., Garderet L. (2007). Genetic abnormalities and survival in multiple myeloma: The experience of the Intergroupe Francophone du Myélome. Blood.

[66] Walker B.A., Leone P.E., Chiecchio L., Dickens N.J., Jenner M.W., Boyd K.D., Johnson D.C., Gonzalez D., Dagrada G.P., Protheroe R.K.M. (2010). A compendium of myeloma-associated chromosomal copy number abnormalities and their prognostic value. Blood.

[67] Bergsagel P.L., Chesi M.V. (2013). Molecular classification and risk stratification of myeloma. Hematol. Oncol..

[68] Shah M.Y., Martinez-Garcia E., Phillip J.M., Chambliss A.B., Popovic R., Ezponda T., Small E.C., Will C., Phillip M.P., Neri P. (2016). MMSET/WHSC1 enhances DNA damage repair leading to an increase in resistance to chemotherapeutic agents. Oncogene.

[69] Martinez-Garcia E., Popovic R., Min D.-J., Sweet S.M.M., Thomas P.M., Zamdborg L., Heffner A., Will C., Lamy L., Staudt L.M. (2011). The MMSET histone methyl transferase switches global histone methylation and alters gene expression in t(4;14) multiple myeloma cells. Blood.

[70] Popovic R., Martinez-Garcia E., Giannopoulou E.G., Zhang Q., Zhang Q., Ezponda T., Shah M.Y., Zheng Y., Will C.M., Small E.C. (2014). Histone methyltransferase MMSET/NSD2 alters EZH2 binding and reprograms the myeloma epigenome through global and focal changes in H3K36 and H3K27 methylation. PLoS Genet..

[71] Xie Z., Bi C., Chooi J.Y., Chan Z.L., Mustafa N., Chng W.J. (2015). MMSET regulates expression of IRF4 in t(4;14) myeloma and its silencing potentiates the effect of bortezomib. Leukemia.

[72] Park J.W., Chae Y.-C., Kim J.-Y., Oh H., Seo S.-B. (2018). Methylation of Aurora kinase A by MMSET reduces p53 stability and regulates cell proliferation and apoptosis. Oncogene.

[73] Min D.-J., Ezponda T., Kim M.K., Will C.M., Martinez-Garcia E., Popovic R., Basrur V., Elenitoba-Johnson K.S., Licht J.D. (2013). MMSET stimulates myeloma cell growth through microRNA-mediated modulation of c-MYC. Leukemia.

[74] Marango J., Shimoyama M., Nishio H., Meyer J.A., Min D.-J., Sirulnik A., Martinez-Martinez Y., Chesi M., Bergsagel P.L., Zhou M.-M. (2008). The MMSET protein is a histone methyltransferase with characteristics of a transcriptional corepressor. Blood.

[75] Pei H., Zhang L., Luo K., Qin Y., Chesi M., Fei F., Bergsagel P.L., Wang L., You Z., Lou Z. (2011). MMSET regulates histone H4K20 methylation and 53BP1 accumulation at DNA damage sites. Nature.

[76] Brito J.L.R., Walker B., Jenner M., Dickens N.J., Brown N.J.M., Ross F.M., Avramidou A., Irving J.A.E., Gonzalez D., Davies F.E. (2009). MMSET deregulation affects cell cycle progression and adhesion regulons in t(4;14) myeloma plasma cells. Haematologica.

[77] Kuo A.J., Cheung P., Chen K., Zee B.M., Kioi M., Lauring J., Xi Y., Park B.H., Shi X., Garcia B.A. (2011). NSD2 links dimethylation of histone H3 at lysine 36 to oncogenic programming. Mol. Cell.

[78] Mason M.J., Schinke C., Eng C.L.P., Towfic F., Gruber F., Dervan A., White B.S., Pratapa A., Guan Y., Chen H. (2020). Multiple Myeloma DREAM Challenge reveals epigenetic regulator PHF19 as marker of aggressive disease. Leukemia.

[79] Gullà A., Hideshima T., Bianchi G., Fulciniti M., Kemal Samur M., Qi J., Tai Y.-T., Harada T., Morelli E., Amodio N. (2018). Protein arginine methyltransferase 5 has prognostic relevance and is a druggable target in multiple myeloma. Leukemia.

[80] Chapman M.A., Brunet J.-P., Keats J.J., Baker A., Adli M., Schinzel A.C., Ahmann G., Christina H., Moore A., Shanmugam V. (2009). HOXA9 Is a Novel Therapeutic Target in Multiple Myeloma. Blood.

[81] Garcia-Gomez A., Li T., de la Calle-Fabregat C., Rodríguez-Ubreva J., Ciudad L., Català-Moll F., Godoy-Tena G., Martín-Sánchez M., San-Segundo L., Muntión S. (2021). Targeting aberrant DNA methylation in mesenchymal stromal cells as a treatment for myeloma bone disease. Nat. Commun..

[82] Herviou L., Cavalli G., Cartron G., Klein B., Moreaux J. (2015). EZH2 in normal hematopoiesis and hematological malignancies. Oncotarget.

[83] Pawlyn C., Bright M.D., Buros A.F., Stein C.K., Walters Z., Aronson L.I., Mirabella F., Jones J.R., Kaiser M.F., Walker B.A. (2017). Overexpression of EZH2 in multiple myeloma is associated with poor prognosis and dysregulation of cell cycle control. Blood Cancer J..

[84] Croonquist P.A., Van Ness B. (2005). The polycomb group protein enhancer of zeste homolog 2 (EZH2) is an oncogene that influences myeloma cell growth and the mutant ras phenotype. Oncogene.

[85] Alzrigat M., Párraga A.A., Agarwal P., Zureigat H., Österborg A., Nahi H., Ma A., Jin J., Nilsson K., Öberg F. (2016). EZH2 inhibition in multiple myeloma downregulates myeloma associated oncogenes and upregulates microRNAs with potential tumor suppressor functions. Oncotarget.

[86] Ishiguro K., Kitajima H., Niinuma T., Maruyama R., Nishiyama N., Ohtani H., Sudo G., Toyota M., Sasaki H., Yamamoto E. (2021). Dual EZH2 and G9a inhibition suppresses multiple myeloma cell proliferation by regulating the interferon signal and IRF4-MYC axis. Cell Death Discov..

[87] Ishiguro K., Kitajima H., Niinuma T., Ishida T., Maruyama R., Ikeda H., Hayashi T., Sasaki H., Wakasugi H., Nishiyama K. (2019). DOT1L inhibition blocks multiple myeloma cell proliferation by suppressing IRF4-MYC signaling. Haematologica.

[88] Dafflon C., Gaulis S., Barys L., Kapur K., Cornacchione V., Schukur L., Bergling S., Traggiai E., Jansky S., Hellmann L. (2020). DOT1L inhibition is lethal for multiple myeloma due to perturbation of the endoplasmic reticulum stress pathway. Oncotarget.

[89] Wei X., Calvo-Vidal M.N., Chen S., Wu G., Revuelta M.V., Sun J., Zhang J., Walsh M.F., Nichols K.E., Joseph V. (2018). Germline Lysine-Specific Demethylase 1 (LSD1/KDM1A) Mutations Confer Susceptibility to Multiple Myeloma. Cancer Res..

[90] Ohguchi H., Harada T., Sagawa M., Kikuchi S., Tai Y.-T., Richardson P.G., Hideshima T., Anderson K.C. (2017). KDM6B modulates MAPK pathway mediating multiple myeloma cell growth and survival. Leukemia.

[91] Ezponda T., Dupéré-Richer D., Will C.M., Small E.C., Varghese N., Patel T., Nabet B., Popovic R., Oyer J., Bulic M. (2017). UTX/KDM6A Loss Enhances the Malignant Phenotype of Multiple Myeloma and Sensitizes Cells to EZH2 inhibition. Cell Rep..

[92] Ohguchi H., Hideshima T., Bhasin M.K., Gorgun G.T., Santo L., Cea M., Samur M.K., Mimura N., Suzuki R., Tai Y.-T. (2016). The KDM3A-KLF2-IRF4 axis maintains myeloma cell survival. Nat. Commun..

[93] Tumber A., Nuzzi A., Hookway E.S., Hatch S.B., Velupillai S., Johansson C., Kawamura A., Savitsky P., Yapp C., Szykowska A. (2017). Potent and Selective KDM5 Inhibitor Stops Cellular Demethylation of H3K4me3 at Transcription Start Sites and Proliferation of MM1S Myeloma Cells. Cell Chem. Biol..

[94] Agarwal P., Alzrigat M., Párraga A.A., Enroth S., Singh U., Ungerstedt J., Österborg A., Brown P.J., Ma A., Jin J. (2016). Genome-wide profiling of histone H3 lysine 27 and lysine 4 trimethylation in multiple myeloma reveals the importance of Polycomb gene targeting and highlights EZH2 as a potential therapeutic target. Oncotarget.

[95] Kalushkova A., Fryknäs M., Lemaire M., Fristedt C., Agarwal P., Eriksson M., Deleu S., Atadja P., Osterborg A., Nilsson K. (2010). Polycomb target genes are silenced in multiple myeloma. PLoS ONE.

[96] Walker B.A., Mavrommatis K., Wardell C.P., Ashby T.C., Bauer M., Davies F.E., Rosenthal A., Wang H., Qu P., Hoering A. (2018). Identification of novel mutational drivers reveals oncogene dependencies in multiple myeloma. Blood.

[97] Yuan L.W., Gambee J.E. (2001). Histone acetylation by p300 is involved in CREB-mediated transcription on chromatin. Biochim. Biophys. Acta.

[98] Dutta R., Tiu B., Sakamoto K.M. (2016). CBP/p300 acetyltransferase activity in hematologic malignancies. Mol. Genet. Metab..

[99] Haberland M., Montgomery R.L., Olson E.N. (2009). The many roles of histone deacetylases in development and physiology: Implications for disease and therapy. Nat. Rev. Genet..

[100] Caprio C., Sacco A., Giustini V., Roccaro A.M. (2020). Epigenetic Aberrations in Multiple Myeloma. Cancers.

[101] Rahman S., Sowa M.E., Ottinger M., Smith J.A., Shi Y., Harper J.W., Howley P.M. (2011). The Brd4 Extraterminal Domain Confers Transcription Activation Independent of pTEFb by Recruiting Multiple Proteins, Including NSD3. Mol. Cell. Biol..

[102] Filippakopoulos P., Qi J., Picaud S., Shen Y., Smith W.B., Fedorov O., Morse E.M., Keates T., Hickman T.T., Felletar I. (2010). Selective inhibition of BET bromodomains. Nature.

[103] Frye S.V. (2010). The art of the chemical probe. Nat. Chem. Biol..

[104] Delmore J.E., Issa G.C., Lemieux M.E., Rahl P.B., Shi J., Jacobs H.M., Kastritis E., Gilpatrick T., Paranal R.M., Qi J. (2011). BET bromodomain inhibition as a therapeutic strategy to target c-Myc. Cell.

[105] Rahl P.B., Lin C.Y., Seila A.C., Flynn R.A., McCuine S., Burge C.B., Sharp P.A., Young R.A. (2010). c-Myc regulates transcriptional pause release. Cell.

[106] Sive J.I., Feber A., Smith D., Quinn J., Beck S., Yong K. (2016). Global hypomethylation in myeloma is associated with poor prognosis. Br. J. Haematol..

[107] Igarashi S., Suzuki H., Niinuma T., Shimizu H., Nojima M., Iwaki H., Nobuoka T., Nishida T., Miyazaki Y., Takamaru H. (2010). A novel correlation between LINE-1 hypomethylation and the malignancy of gastrointestinal stromal tumors. Clin. Cancer Res..

[108] Daskalos A., Nikolaidis G., Xinarianos G., Savvari P., Cassidy A., Zakopoulou R., Kotsinas A., Gorgoulis V., Field J.K., Liloglou T. (2009). Hypomethylation of retrotransposable elements correlates with genomic instability in non-small cell lung cancer. Int. J. Cancer.

[109] Wilop S., van Gemmeren T.B., Lentjes M.H.F.M., van Engeland M., Herman J.G., Brümmendorf T.H., Jost E., Galm O. (2011). Methylation-associated dysregulation of the suppressor of cytokine signaling-3 gene in multiple myeloma. Epigenetics.

[110] Avet-Loiseau H., Leleu X., Roussel M., Moreau P., Guerin-Charbonnel C., Caillot D., Marit G., Benboubker L., Voillat L., Mathiot C. (2010). Bortezomib plus dexamethasone induction improves outcome of patients with t(4;14) myeloma but not outcome of patients with del(17p). J. Clin. Oncol..

[111] Schroeder M.A., Fiala M.A., Ghobadi A., Stockerl-Goldstein K.E., Wildes T.M., Vij R. (2017). Overexpression of EZH2 in Multiple Myeloma Is Associated with Poor Prognosis Regardless of Treatment with Novel Agents or High-Dose Chemotherapy. Blood.

[112] Mithraprabhu S., Kalff A., Chow A., Khong T., Spencer A. (2014). Dysregulated Class I histone deacetylases are indicators of poor prognosis in multiple myeloma. Epigenetics.

[113] Adamik J., Roodman G.D., Galson D.L. (2019). Epigenetic-Based Mechanisms of Osteoblast Suppression in Multiple Myeloma Bone Disease. JBMR Plus.

[114] Adamik J., Jin S., Sun Q., Zhang P., Weiss K.R., Anderson J.L., Silbermann R., Roodman G.D., Galson D.L. (2017). EZH2 or HDAC1 Inhibition Reverses Multiple Myeloma–Induced Epigenetic Suppression of Osteoblast Differentiation. Mol. Cancer Res..

[115] Adamik J., Silbermann R., Marino S., Sun Q., Anderson J.L., Zhou D., Xie X.-Q., Roodman G.D., Galson D.L. (2018). XRK3F2 Inhibition of p62-ZZ Domain Signaling Rescues Myeloma-Induced GFI1-Driven Epigenetic Repression of the Runx2 Gene in Pre-osteoblasts to Overcome Differentiation Suppression. Front. Endocrinol..

[116] Teramachi J., Silbermann R., Yang P., Zhao W., Mohammad K.S., Guo J., Anderson J.L., Zhou D., Feng R., Myint K.-Z. (2016). Blocking the ZZ domain of sequestosome1/p62 suppresses myeloma growth and osteoclast formation in vitro and induces dramatic bone formation in myeloma-bearing bones in vivo. Leukemia.

[117] Kikuchi J., Koyama D., Wada T., Izumi T., Hofgaard P.O., Bogen B., Furukawa Y. (2015). Phosphorylation-mediated EZH2 inactivation promotes drug resistance in multiple myeloma. J. Clin. Investig..

[118] Abdi J., Rastgoo N., Chen Y., Chen G.A., Chang H. (2019). Ectopic expression of BIRC5-targeting miR-101-3p overcomes bone marrow stroma-mediated drug resistance in multiple myeloma cells. BMC Cancer.

[119] Ho M., Chen T., Liu J., Dowling P., Hideshima T., Zhang L., Morelli E., Camci-Unal G., Wu X., Tai Y.-T. (2020). Targeting histone deacetylase 3 (HDAC3) in the bone marrow microenvironment inhibits multiple myeloma proliferation by modulating exosomes and IL-6 trans-signaling. Leukemia.

[120] Maes K., Menu E., Van Valckenborgh E., Van Riet I., Vanderkerken K., De Bruyne E. (2013). Epigenetic Modulating Agents as a New Therapeutic Approach in Multiple Myeloma. Cancers.

[121] Todoerti K., Barbui V., Pedrini O., Lionetti M., Fossati G., Mascagni P., Rambaldi A., Neri A., Introna M., Lombardi L. (2010). Pleiotropic anti-myeloma activity of ITF2357: Inhibition of interleukin-6 receptor signaling and repression of miR-19a and miR-19b. Haematologica.

[122] Mitsiades C.S., Mitsiades N.S., McMullan C.J., Poulaki V., Shringarpure R., Hideshima T., Akiyama M., Chauhan D., Munshi N., Gu X. (2004). Transcriptional signature of histone deacetylase inhibition in multiple myeloma: Biological and clinical implications. Proc. Natl. Acad. Sci. USA.

[123] Oike Y., Takakura N., Hata A., Kaname T., Akizuki M., Yamaguchi Y., Yasue H., Araki K., Yamamura K., Suda T. (1999). Mice homozygous for a truncated form of CREB-binding protein exhibit defects in hematopoiesis and vasculo-angiogenesis. Blood.

[124] Chmelarova M., Palicka V. (2019). Epigenetics in cancer: A promising path to follow?. Clin. Chem. Lab. Med..

[125] Imai Y., Hirano M., Kobayashi M., Futami M., Tojo A. (2019). HDAC Inhibitors Exert Anti-Myeloma Effects through Multiple Modes of Action. Cancers.

[126] Wolf J.L., Siegel D., Goldschmidt H., Hazell K., Bourquelot P.M., Bengoudifa B.R., Matous J., Vij R., de Magalhaes-Silverman M., Abonour R. (2012). Phase II trial of the pan-deacetylase inhibitor panobinostat as a single agent in advanced relapsed/refractory multiple myeloma. Leuk. Lymphoma.

[127] Ocio E.M., Vilanova D., Atadja P., Maiso P., Crusoe E., Fernández-Lázaro D., Garayoa M., San-Segundo L., Hernández-Iglesias T., de Alava E. (2010). In vitro and in vivo rationale for the triple combination of panobinostat (LBH589) and dexamethasone with either bortezomib or lenalidomide in multiple myeloma. Haematologica.

[128] Kikuchi J., Wada T., Shimizu R., Izumi T., Akutsu M., Mitsunaga K., Noborio-Hatano K., Nobuyoshi M., Ozawa K., Kano Y. (2010). Histone deacetylases are critical targets of bortezomib-induced cytotoxicity in multiple myeloma. Blood.

[129] Raedler L.A. (2016). Farydak (Panobinostat): First HDAC Inhibitor Approved for Patients with Relapsed Multiple Myeloma. Am. Health Drug Benefits.

[130] European Medicines Agency. https://www.ema.europa.eu/en/documents/assessment-report/farydak-epar-public-assessment-report_en.pdf.

[131] San-Miguel J.F., Hungria V.T.M., Yoon S.-S., Beksac M., Dimopoulos M.A., Elghandour A., Jedrzejczak W.W., Günther A., Nakorn T.N., Siritanaratkul N. (2014). Panobinostat plus bortezomib and dexamethasone versus placebo plus bortezomib and dexamethasone in patients with relapsed or relapsed and refractory multiple myeloma: A multicentre, randomised, double-blind phase 3 trial. Lancet Oncol..

[132] San Miguel J.F., Hungria V.T.M., Yoon S.-S., Beksac M., Dimopoulos M.A., Elghandour A., Jedrzejczak W.W., Guenther A., Na Nakorn T., Siritanaratkul N. (2015). Panobinostat plus bortezomib and dexamethasone in patients with relapsed or relapsed and refractory multiple myeloma who received prior bortezomib and IMiDs: A predefined subgroup analysis of PANORAMA. J. Clin. Oncol..

[133] San-Miguel J.F., Hungria V.T.M., Yoon S.-S., Beksac M., Dimopoulos M.A., Elghandour A., Jedrzejczak W.W., Günther A., Nakorn T.N., Siritanaratkul N. (2016). Overall survival of patients with relapsed multiple myeloma treated with panobinostat or placebo plus bortezomib and dexamethasone (the PANORAMA 1 trial): A randomised, placebo-controlled, phase 3 trial. Lancet Haematol..

[134] Yee A.J., Raje N.S. (2018). Panobinostat and Multiple Myeloma in 2018. Oncologist.

[135] Laubach J.P., Schjesvold F., Mariz M., Dimopoulos M.A., Lech-Maranda E., Spicka I., Hungria V.T.M., Shelekhova T., Abdo A., Jacobasch L. (2021). Efficacy and safety of oral panobinostat plus subcutaneous bortezomib and oral dexamethasone in patients with relapsed or relapsed and refractory multiple myeloma (PANORAMA 3): An open-label, randomised, phase 2 study. Lancet Oncol..

[136] Chari A., Cho H.J., Dhadwal A., Morgan G., La L., Zarychta K., Catamero D., Florendo E., Stevens N., Verina D. (2017). A phase 2 study of panobinostat with lenalidomide and weekly dexamethasone in myeloma. Blood Adv..

[137] Berdeja J.G., Gregory T.K., Faber E.A., Hart L.L., Mace J.R., Arrowsmith E.R., Flinn I.W., Matous J.V. (2021). A phase I/II study of the combination of panobinostat and carfilzomib in patients with relapsed or relapsed/refractory multiple myeloma: Final analysis of second dose-expansion cohort. Am. J. Hematol..

[138] Kaufman J.L., Mina R., Shah J.J., Laubach J.P., Nooka A.K., Lewis C., Gleason C., Sharp C., Harvey R.D., Heffner L.T. (2020). Phase 1 Trial Evaluating Vorinostat Plus Bortezomib, Lenalidomide, and Dexamethasone in Patients With Newly Diagnosed Multiple Myeloma. Clin. Lymphoma Myeloma Leuk..

[139] Manasanch E.E., Shah J.J., Lee H.C., Weber D.M., Thomas S.K., Amini B., Feng L., Berkova Z., Hildebrandt M., Orlowski R.Z. (2018). Bortezomib, lenalidomide, and dexamethasone with panobinostat for front-line treatment of patients with multiple myeloma who are eligible for transplantation: A phase 1 trial. Lancet Haematol..

[140] Berenson J.R., Hilger J.D., Yellin O., Boccia R.V., Matous J., Dressler K., Ghazal H.H., Jamshed S., Kingsley E.C., Harb W.A. (2014). A phase 1/2 study of oral panobinostat combined with melphalan for patients with relapsed or refractory multiple myeloma. Ann. Hematol..

[141] Richardson P.G., Schlossman R.L., Alsina M., Weber D.M., Coutre S.E., Gasparetto C., Mukhopadhyay S., Ondovik M.S., Khan M., Paley C.S. (2013). PANORAMA 2: Panobinostat in combination with bortezomib and dexamethasone in patients with relapsed and bortezomib-refractory myeloma. Blood.

[142] Suzuki K., Sunami K., Matsumoto M., Maki A., Shimada F., Suzuki K., Shimizu K. (2021). Phase II, Multicenter, Single-Arm, Open-Label Study to Evaluate the Efficacy and Safety of Panobinostat in Combination with Bortezomib and Dexamethasone in Japanese Patients with Relapsed or Relapsed-and-Refractory Multiple Myeloma. Acta Haematol..

[143] Mitsiades N., Mitsiades C.S., Richardson P.G., McMullan C., Poulaki V., Fanourakis G., Schlossman R., Chauhan D., Munshi N.C., Hideshima T. (2003). Molecular sequelae of histone deacetylase inhibition in human malignant B cells. Blood.

[144] Kelly W.K., Marks P.A. (2005). Drug insight: Histone deacetylase inhibitors--development of the new targeted anticancer agent suberoylanilide hydroxamic acid. Nat. Clin. Pract. Oncol..

[145] Pei X.-Y., Dai Y., Grant S. (2004). Synergistic Induction of Oxidative Injury and Apoptosis in Human Multiple Myeloma Cells by the Proteasome Inhibitor Bortezomib and Histone Deacetylase Inhibitors. Clin. Cancer Res..

[146] Dimopoulos M., Siegel D.S., Lonial S., Qi J., Hajek R., Facon T., Rosinol L., Williams C., Blacklock H., Goldschmidt H. (2013). Vorinostat or placebo in combination with bortezomib in patients with multiple myeloma (VANTAGE 088): A multicentre, randomised, double-blind study. Lancet Oncol..

[147] Brown S., Pawlyn C., Tillotson A.-L., Sherratt D., Flanagan L., Low E., Morgan G.J., Williams C., Kaiser M., Davies F.E. (2021). Bortezomib, Vorinostat, and Dexamethasone Combination Therapy in Relapsed Myeloma: Results of the Phase 2 MUK four Trial. Clin. Lymphoma Myeloma Leuk..

[148] Sborov D.W., Benson D.M., Williams N., Huang Y., Bowers M.A., Humphries K., Efebera Y., Devine S., Hofmeister C.C. (2015). Lenalidomide and vorinostat maintenance after autologous transplant in multiple myeloma. Br. J. Haematol..

[149] Holmberg L.A., Green D., Libby E., Becker P.S. (2020). Bortezomib and Vorinostat Therapy as Maintenance Therapy after Autologous Transplant for Multiple Myeloma. Acta Haematol..

[150] Niesvizky R., Ely S., Mark T., Aggarwal S., Gabrilove J.L., Wright J.J., Chen-Kiang S., Sparano J.A. (2011). Phase 2 trial of the histone deacetylase inhibitor romidepsin for the treatment of refractory multiple myeloma. Cancer.

[151] Galli M., Salmoiraghi S., Golay J., Gozzini A., Crippa C., Pescosta N., Rambaldi A. (2010). A phase II multiple dose clinical trial of histone deacetylase inhibitor ITF2357 in patients with relapsed or progressive multiple myeloma. Ann. Hematol..

[152] Mishima Y., Santo L., Eda H., Cirstea D., Nemani N., Yee A.J., O’Donnell E., Selig M.K., Quayle S.N., Arastu-Kapur S. (2015). Ricolinostat (ACY-1215) induced inhibition of aggresome formation accelerates carfilzomib-induced multiple myeloma cell death. Br. J. Haematol..

[153] Vogl D.T., Raje N., Jagannath S., Richardson P., Hari P., Orlowski R., Supko J.G., Tamang D., Yang M., Jones S.S. (2017). Ricolinostat, the first selective histone deacetylase 6 inhibitor, in combination with bortezomib and dexamethasone for relapsed or refractory multiple myeloma. Clin. Cancer Res..

[154] Niesvizky R., Richardson P.G., Gabrail N.Y., Madan S., Yee A.J., Quayle S.N., Almeciga-Pinto I., Jones S.S., Houston L., Hayes D. (2015). ACY-241, a Novel, HDAC6 Selective Inhibitor: Synergy with Immunomodulatory (IMiD^®^) Drugs in Multiple Myeloma (MM) Cells and Early Clinical Results (ACE-MM-200 Study). Blood.

[155] Won H.-R., Lee D.H., Yeon S.-K., Ryu H.-W., Kim G.W., Kwon S.H. (2019). HDAC6-selective inhibitor synergistically enhances the anticancer activity of immunomodulatory drugs in multiple myeloma. Int. J. Oncol..

[156] Stamato M.A., Juli G., Romeo E., Ronchetti D., Arbitrio M., Caracciolo D., Neri A., Tagliaferri P., Tassone P., Amodio N. (2017). Inhibition of EZH2 triggers the tumor suppressive miR-29b network in multiple myeloma. Oncotarget.

[157] Harada T., Hideshima T., Anderson K.C. (2016). Histone deacetylase inhibitors in multiple myeloma: From bench to bedside. Int. J. Hematol..

[158] Juli G., Oliverio M., Bellizzi D., Gallo Cantafio M.E., Grillone K., Passarino G., Colica C., Nardi M., Rossi M., Procopio A. (2019). Anti-tumor Activity and Epigenetic Impact of the Polyphenol Oleacein in Multiple Myeloma. Cancers.

[159] Yao R., Han D., Sun X., Xie Y., Wu Q., Fu C., Yao Y., Li H., Li Z., Xu K. (2018). Scriptaid inhibits cell survival, cell cycle, and promotes apoptosis in multiple myeloma via epigenetic regulation of p21. Exp. Hematol..

[160] Turner J.G., Gump J.L., Zhang C., Cook J.M., Marchion D., Hazlehurst L., Munster P., Schell M.J., Dalton W.S., Sullivan D.M. (2006). ABCG2 expression, function, and promoter methylation in human multiple myeloma. Blood.

[161] Dimopoulos K., Søgaard Helbo A., Fibiger Munch-Petersen H., Sjö L., Christensen J., Sommer Kristensen L., Asmar F., Hermansen N.E.U., O’Connel C., Gimsing P. (2018). Dual inhibition of DNMTs and EZH2 can overcome both intrinsic and acquired resistance of myeloma cells to IMiDs in a cereblon-independent manner. Mol. Oncol..

[162] Harding T., Swanson J., Van Ness B. (2018). EZH2 inhibitors sensitize myeloma cell lines to panobinostat resulting in unique combinatorial transcriptomic changes. Oncotarget.

[163] Zeng D., Liu M., Pan J. (2017). Blocking EZH2 methylation transferase activity by GSK126 decreases stem cell-like myeloma cells. Oncotarget.

[164] Yuan X.-G., Huang Y.-R., Yu T., Jiang H.-W., Xu Y., Zhao X.-Y. (2019). Chidamide, a histone deacetylase inhibitor, induces growth arrest and apoptosis in multiple myeloma cells in a caspase-dependent manner. Oncol. Lett..

[165] Das D.S., Ray A., Das A., Song Y., Tian Z., Oronsky B., Richardson P., Scicinski J., Chauhan D., Anderson K.C. (2016). A novel hypoxia-selective epigenetic agent RRx-001 triggers apoptosis and overcomes drug resistance in multiple myeloma cells. Leukemia.

[166] Kiziltepe T., Hideshima T., Catley L., Raje N., Yasui H., Shiraishi N., Okawa Y., Ikeda H., Vallet S., Pozzi S. (2007). 5-Azacytidine, a DNA methyltransferase inhibitor, induces ATR-mediated DNA double-strand break responses, apoptosis, and synergistic cytotoxicity with doxorubicin and bortezomib against multiple myeloma cells. Mol. Cancer Ther..

[167] Nojima M., Maruyama R., Yasui H., Suzuki H., Maruyama Y., Tarasawa I., Sasaki Y., Asaoku H., Sakai H., Hayashi T. (2009). Genomic Screening for Genes Silenced by DNA Methylation Revealed an Association between RASD1 Inactivation and Dexamethasone Resistance in Multiple Myeloma. Clin. Cancer Res..

[168] Vincenz L., Jäger R., O’Dwyer M., Samali A. (2013). Endoplasmic reticulum stress and the unfolded protein response: Targeting the Achilles heel of multiple myeloma. Mol. Cancer Ther..

[169] Ocio E.M., San Miguel J.F. (2010). The DAC system and associations with multiple myeloma. Investig. New Drugs.

